# Effective population size and the genetic consequences of commercial whaling on the humpback whales (*Megaptera novaeangliae*) from Southwestern Atlantic Ocean

**DOI:** 10.1590/1678-4685-GMB-2017-0052

**Published:** 2018

**Authors:** Ana Lúcia Cypriano-Souza, Tiago Ferraz da Silva, Márcia H. Engel, Sandro L. Bonatto

**Affiliations:** 1 Faculdade de Biociências, Pontifícia Universidade Católica do Rio Grande do Sul (PUCRS), Porto Alegre, RS, Brazil; 2 Projeto Baleia Jubarte/Instituto Baleia Jubarte, Caravelas, BA, Brazil; 3 Departamento de Genética e Biologia Evolutiva, Instituto de Biociências, Universidade de São Paulo (USP), São Paulo, SP, Brazil

**Keywords:** Commercial whaling, bottleneck, humpback whale, microsatellites, demography

## Abstract

Genotypes of 10 microsatellite loci of 420 humpback whales from the Southwestern Atlantic Ocean population were used to estimate for the first time its contemporary effective (*N*_*e*_) and census (*N*_*c*_) population sizes and to test the genetic effect of commercial whaling. The results are in agreement with our previous studies that found high genetic diversity for this breeding population. Using an approximate Bayesian computation approach, the scenario of constant *N*_*e*_ was significantly supported over scenarios with moderate to strong size changes during the commercial whaling period. The previous generation *N*_*c*_ (*N*_*e*_ multiplied by 3.6), which should corresponds to the years between around 1980 and 1990, was estimated between ~2,600 and 6,800 whales (point estimate ~4,000), and is broadly compatible with the recent abundance surveys extrapolated to the past using a growth rate of 7.4% per annum. The long-term *N*_*c*_ in the constant scenario (point estimate ~15,000) was broadly compatible (considering the confidence interval) with pre-whaling catch records estimates (point estimate ~25,000). Overall, our results shown that the Southwestern Atlantic Ocean humpback whale population is genetically very diverse and resisted well to the strong population reduction during commercial whaling.

## Introduction

Estimation of historic (pre-whaling) and contemporary population sizes are important to offer guidelines for managing and restoring populations that suffered overexploitation, such as those of baleen whales (Baker and Clapham, 2004; Jackson *et al.*, 2008). Although abundance is obviously important in the very short term, effective population size (*N*_*e*_) is a key parameter in the long term, and essential for population genetics, evolutionary biology and conservation biology (Schwartz *et al.*, 1998; Charlesworth, 2009). It is directly related to evolutionary processes, such as rates of genetic drift and loss of genetic variability, levels of inbreeding, and effectiveness of selection (Frankham *et al.*, 2002). Generally, *N*_*e*_ is lower than the census population size (*N*_*c*_) since individuals do not contribute genes equally to the next generation. One important advance has been the development of methods to estimate *N*_*e*_ from genetic data (Schwartz *et al.*, 1998; Leberg, 2005; Waples, 2005; Palstra and Ruzzante, 2008; Luikart *et al.*, 2010). This approach has provided important information to investigate whale population dynamics, although *N*_*e*_ estimates are only available for some species and in a few areas (e.g. Rooney *et al.*, 1999, 2001; Waldick *et al.*, 2002; Roman and Palumbi, 2003; Alter *et al.*, 2007, 2012; Ruegg *et al.*, 2010, 2013). Another relevant information for management is knowing the impact induced by the extreme reduction of the abundance during 20^th^ century whaling activities on the genetic diversity of the species, since the loss of genetic variation can impact population viability leading to its premature extinction. However, although conservation actions should prioritize the populations with minor genetic diversity, it is not known which populations went extinct following such bottlenecks.

Humpback whales, *Megaptera novaeangliae* (Borowski 1781), were among the most exploited baleen whale species by commercial whaling. The populations are found throughout the world’s ocean basins, undertaking annual migrations between the low latitude waters, where they breed and calve during the winter-spring months, and the high latitude waters, where they feed during the summer (Dawbin, 1966). The International Whaling Commission (IWC) recognizes seven humpback whale breeding stocks (termed A-G) in the Southern Hemisphere (International Whaling Commission, 2015). In the Southwestern Atlantic Ocean, the humpback whale population wintering along the Brazilian coast (~ 5° to 23° S) (Andriolo *et al.*, 2010) is recognized as the Breeding Stock A (BSA). The main mating and calving area for this population is in the Abrolhos Bank (16°40’- 19°30’ S and 37°25’- 39°45’ W), where most whales concentrate (about 85% of the density) during the breeding season (Andriolo *et al.*, 2010; Bortolotto *et al.*, 2016a). However, in recent years, the number of sightings and strandings has increased beyond the BSA range, indicating recovery of this population and likely expansion of its distribution range (Pretto *et al.*, 2009; Wedekin *et al.*, 2014; Bortolotto *et al.*, 2016b). This population migrates to summer feeding grounds around South Georgia and South Sandwich islands in the Southern Ocean (Engel and Martin, 2009; Zerbini *et al.*, 2006, 2011a).

Commercial whaling during the 20^th^ century reduced the worldwide humpback whale population to a small fraction of its pre-exploitation abundance (Tønnessen and Johnsen, 1982). In the Southern Hemisphere, approximately 200,000 humpback whales were hunted from 1904 to 1972, after accounting for the illegal Soviet whaling, mainly by whaling operations around Antarctica feeding areas (Findlay, 2001; Clapham *et al.*, 2009; Allison, 2010). In Brazil, pre-modern whaling began in the early 1600s, ending in the 1830s in southern of the country, with the collapse of the southern right whale population, but lasted until the 1920s in northeastern region due to the high density of humpback whales, mainly in Caravelas, Bahia. It was estimated that between 11,000 and 32,000 humpback whales were captured from 1830 to 1924 (Morais *et al.*, 2017). Modern whaling operations that began in the 20^th^ century expanded the activities of the whaling stations mainly for the coasts of Costinha (7° S) (between 1910 and 1967) and Cabo Frio (23° S) (between 1960 and 1963), where 352 humpbacks were caught in 1913, but only around 13 whales in 1967, already indicating a significant population size reduction (Paiva and Grangeiro, 1965, 1970; Williamson, 1975). In addition, modern whaling activities in high-density areas in the Antarctic and Sub-Antarctic feeding grounds increased the annual catch to several thousand whales (Findlay, 2001). Only in the surroundings of the South Georgia Island about 22,717 humpback whales were killed between 1904 and 1915, when the exploitation of this stock was most extensive (Edmundson and Hart, 2014). Although the species have been protected since 1966, the former Soviet Union fleet took humpback whales illegally off the central coast of Brazil until 1973 (Yablokov *et al.*, 1998). It was estimated that 48,477 humpbacks were caught by Soviet whaling in the Southern Hemisphere between 1948 and 1973, of which 1,407 were caught in the South Atlantic Ocean between 1960 and 1967 (Berzin, 2008).

The BSA population size before the exploitation by modern whaling was estimated using catch records to nearly 24,700 individuals, and it reached its lowest numbers in the late 1950s, when there were less than 500 individuals (Zerbini *et al.*, 2011b). Presently this population is recovering fast (growth rate of 7.4% per annum, Ward *et al.*, 2011) and the abundance in 2015 was estimated around 12,123 individuals (Pavanato *et al.*, 2017). Interestingly, despite these well-documented census size changes in the BSA, recent studies have not detected a genetic bottleneck, that is, a significant reduction in the effective population size of this population (Engel *et al.*, 2008; Cypriano-Souza *et al.*, 2010). The absence of a signal for a genetic bottleneck was explained by the low intensity (in terms of duration and minimum population size) of this bottleneck (Engel *et al.*, 2008; Cypriano-Souza *et al.*, 2010). However, these studies used standard methods (heterozygosity excess, mode-shift and M-ratio tests), that have reduced power to detect moderate bottlenecks (Peery *et al.*, 2012). Besides, none of the genetic studies so far has provided estimates of *N*_*e*_ for the Brazilian humpback whale population.

The present study aims to estimate the effective and census population sizes of the Southwestern Atlantic Ocean humpback population, and investigate the effects of commercial whaling in the 20^th^ century on its genetic diversity, based on the analysis of genotypes constructed from 10 microsatellite loci for 420 individuals sampled off the Brazilian coast.

## Materials and Methods

### Sample collection and DNA extraction

**Figure 1:**
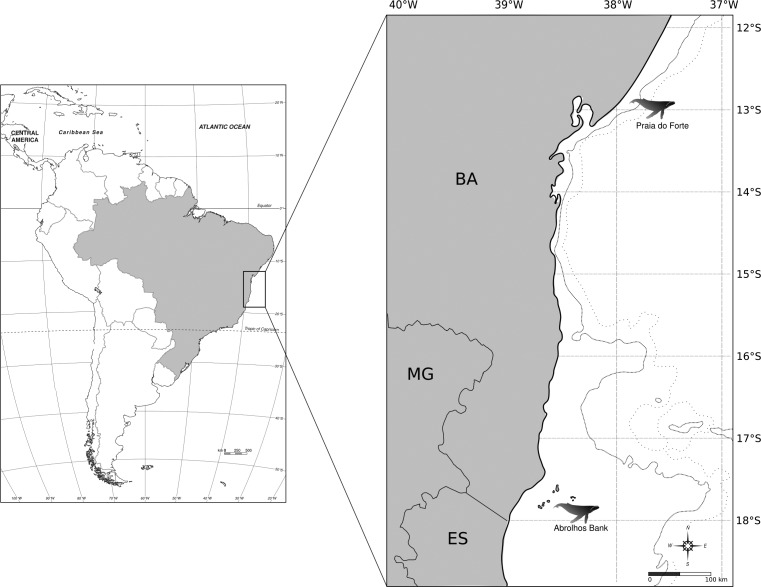
Map of the surveyed areas, showing the geographic locations of the two sampling sites (zoom) of the humpback whale breeding ground off the Brazilian coast (BSA).

Between 1999 and 2007, 379 tissue samples of humpback whales were collected by the biopsy dart procedure (Lambertsen, 1987) at two geographic locations off the Brazilian coast, the Abrolhos Bank (*n* = 332), in southern Bahia and northern Espírito Santo, and Praia do Forte (*n* = 47) in northern Bahia (Figure 1). Only adult animals were sampled within the social groups, which showed a body size longer than 11 meters. Additional samples (*n* = 41) resulted from individuals adults stranded along the coasts of both states. Samples were preserved in 70% ethanol and were stored at -20 °C until processed. Genomic DNA was extracted using proteinase K digestion followed by phenol/chloroform extraction method (Palsbøll *et al.*, 1995) or using the DNeasy Blood and Tissue kit (QIAGEN).

### Microsatellite genotyping

Samples were screened for genetic variation at 10 microsatellite loci [seven dinucleotides: EV1, EV37, EV94, EV96 (Valsecchi and Amos, 1996), 199/200, 417/418, 464/465 (Schlötterer *et al.*, 1991), and three tetranucleotides: GATA028, GATA053, GATA417, (Palsbøll *et al.*, 1997)]. Genotypes of 268 of the individuals used here were described previously in Cypriano-Souza *et al.* (2010), and genotyping of the additional samples was conducted exactly as described in that study, including in the same machine.

### Genetic variation

The program MICRO-CHECKER v.2.2.3 (Van Oosterhout *et al.*, 2004) was used to identify possible null alleles, large allele dropout, and scoring errors due to stutter peaks. While in the previous study (Cypriano-Souza *et al.*, 2010) locus 417/418 showed a weak sign of null alleles, this was not detected here, since homozygous excess was insufficient to suggest the presence of null alleles.

Genetic diversity was measured as the number of alleles per locus (*K*), observed and expected heterozygosities (*H*_*O*_ and *H*_*E*_, respectively) under Hardy-Weinberg equilibrium (HWE) (Nei, 1978), using FSTAT v.2.9.3 (Goudet, 2002). FSTAT was also used to calculate the measure of *F*_*IS*_ (Weir and Cockerham, 1984). Deviations from HWE for each locus (Guo and Thompson, 1992) and linkage disequilibrium between loci were tested using ARLEQUIN 3.5 (Excoffier and Lischer, 2010), corrected for simultaneous comparisons with the sequential Bonferroni test (Rice, 1989).

### N_e_ estimation

Two methods were used to estimate effective population size (*N*_*e*_), both assuming a closed population with discrete generations and random variance in reproductive success. The program NeEstimator v.2.01 (Do *et al.*, 2014) was used to estimate the parental generation *N*_*e*_ from genotypic data based on the linkage disequilibrium (LD) method, which calculates separate estimates using different criteria for excluding rare alleles. We used the random mating model and the following critical values (*P*_*crit*_): 0.05, 0.02 and 0.01, as suggested in the program manual.

**Figure 2:**
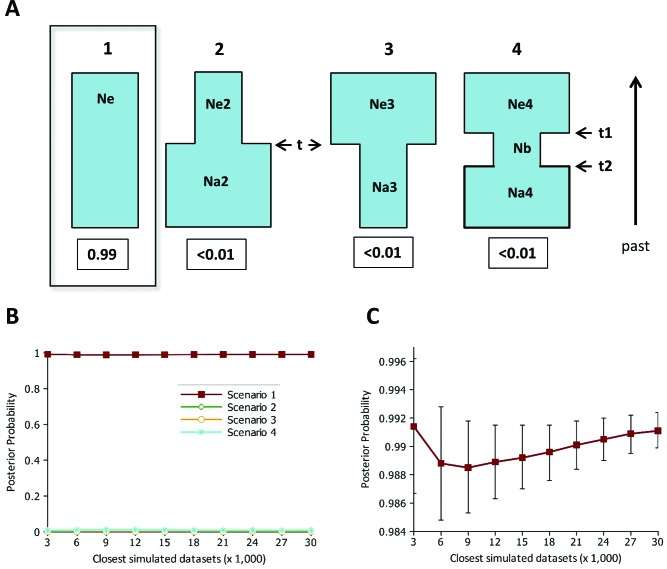
Demographic scenarios for humpback whales from BSA. (a) The four demographic scenarios tested with the DIYABC approach: 1 - constant population, 2 - bottlenecked population, 3 - expanded population, 4 - population with a transitory bottleneck. Demographic parameters: *N*_*e*_ - long-term; *N*_*e*_2, *N*_*e*_3 and *N*_*e*_4 - current; *N*_*a*_2 and *N*_*a*_4 - pre-bottleneck; *N*_*a*_3 and *N*_*b*_ - during bottleneck. The posterior probability of each scenario is given at the bottom. (b) Posterior probabilities (y-axis) with confidence intervals of the four scenarios in different numbers of selected closest-to-observed simulations based on the direct estimate, and (c) logistic regression (only for scenario 1).

An approximate Bayesian computation (ABC) approach implemented in the program DIYABC v.1.0.46 (Cornuet *et al.*, 2008, 2010) was also used to test four different scenarios based in the possible demographic history of this humpback whale population during commercial whaling in the 20^th^ century. Scenario 1 is a constant size population (no bottleneck), scenario 2 consisted of a population that is still experiencing a bottleneck, scenario 3 is a population that expanded recently from a bottleneck, and scenario 4 is a population that experienced a transitory bottleneck (Figure 2a). The priors for all parameters were uniformly distributed between specified minimum and maximum values (Table 1 and Figure 2a), which were based on the available information of the whaling history of this population and its present day census data (see Introduction). The demographic parameters were: Scenario 1: *N*_*e*_ (long-term historical *N*_*e*_); Scenario 2: *N*_*e*_2 (current *N*_*e*_), *N*_*a*_2 (pre-bottleneck *N*_*e*_); Scenario 3: *N*_*e*_ *3* (current *N*_*e*_), *N*_*a*_3 (*N*_*e*_ during bottleneck); *t* (time since demographic change in scenarios 2 and 3); Scenario 4: *N*_*e*_4 (current *N*_*e*_), *N*_*b*_ (*N*_*e*_ during bottleneck), *N*_*a*_ *4* (pre-bottleneck *N*_*e*_), *t2* and *t1* (time since the beginning and end of the bottleneck, respectively). All times are in number of generations [generation time of 18 years taking into account the range between 12 and 24 years estimated for the humpback whales (Chittleborough, 1965; Roman and Palumbi, 2003)] from the present, with *t2* > *t1*. The 10 microsatellites loci were assumed to evolve under the generalized stepwise mutation model (GSM) (Estoup *et al.*, 2002) with the widely used mutation rate (*μ*) range for mammals, from 10^-4^ to 10^-3^ per generation (Ellegren, 1995, Whittaker *et al.*, 2003, Hoffman *et al.*, 2011) and the coefficients of geometric distribution (*P*) from 0.1 to 0.7. Motif sizes and alleles ranges followed the empirical data of each locus. The summary statistics were the mean number of alleles (*A*), genetic diversity (*H*_*E*_), allelic size range (*AR*), and Garza-Williamson’s index (*M*). A total of 3,000,000 simulations were performed to generate the reference table, using the four scenarios according to their prior probability and their parameter values drawn from the prior distributions. The posterior probability of each scenario was assessed using both direct estimate and logistic regression approaches using between 500 and 30,000 best simulations. Under an ABC approach, the best scenario is the one with the simulated data set closest to observed data set. For the best scenario, the posterior distribution of the parameters was estimated using logit transformation for the 8,000 best simulations.

**Table 1:** Prior values (minimum and maximum, with uniform distribution) for the parameters used for the four demographic scenarios (Figure 2a) in the DIYABC approach. Effective sizes are in number of individuals and times are in number of generations (generation time of 18 years).

Scenario/Parameter	Minimum	Maximum
Scenario 1		
*Ne*	10	30,000
Scenario 2		
*Ne2*	10	300
*Na2*	5,000	30,000
*t*	2	10
Scenario 3		
*Ne3*	1,000	5,000
*Na3*	10	300
*t*	2	10
Scenario 4		
*Ne4*	1,000	5,000
*Nb*	10	300
*Na4*	5,000	30,000
*t1* ^*^	2	10
*t2* ^*^	2	10

* *t2 > t1*

We converted the effective sizes (*N*_*e*_) obtained in each method to the census sizes (*N*_*c*_) as follows. First, the ratio of mature adults to the effective number of adults (*N*_*T*_:*N*_*e*_) approaches 2 for most populations, and was based on the equation *N*_*e*_ = *N*/(2 - T^-^1) from Nunney and Elam (1994), where T is the generation length. Second, the proportion of juveniles in the population (number of adults + juveniles)/(number of adults), was estimated between 1.6 to 2.0 for humpback whales based on catch and survey data (Chittleborough, 1965, Roman and Palumbi, 2003). Therefore, multiplying the two ratios, the average ratio of census population size to effective population size was 3.6, with a variation from 3.2 to 4.0, which has also been used in previous studies (Roman and Palumbi, 2003; Alter *et al.*, 2007, 2012; Ruegg *et al.*, 2010, 2013).

## Results

### Genetic variability

Individual multilocus genotypes were on average 98.5% complete. Summary statistics are presented in Table 2. The number of alleles identified at the 10 microsatellite loci ranged from five (EV1) to 18 (GATA417), with a mean of 12.6. The mean observed (*H*_*O*_) heterozygosity was 0.736, ranging from 0.553 (EV1) to 0.923 (GATA417), and the mean expected (*H*_*E*_) heterozygosity was 0.746, ranging from 0.532 (EV1) to 0.923 (EV37). Population-wide *F*_*IS*_ values were low for all loci (below 0.05), except for the locus GATA053 (*F*_*IS*_ = 0.053) and the locus 417/418 (*F*_*IS*_ = 0.068), but these values were not significant. Moreover, no evidence of null alleles and no significant deviation from HWE expectations were seen at any of the loci. Pairwise comparison of allele frequencies revealed no significant linkage disequilibrium after Bonferroni correction.

**Table 2:** Summary statistics for 10 microsatellite loci genotyped for the humpback whale population off Brazil. Rep, repeat motif length in base pairs; *K*, number of alleles; *H*_*O*_, observed heterozygosity; *H*_*E*_, expected heterozygosity; *F*_*IS*_, inbreeding coefficient (*P < 0.005 based on 180 randomizations).

Locus	Rep	Allele range	*K*	*H*_*O*_	*H*_*E*_	*F*_*IS*_
GATA 28	4	143-203	15	0.626	0.612	- 0.022
GATA 53	4	231-287	14	0.791	0.835	0.053
GATA 417	4	186-280	18	0.923	0.909	- 0.016
199/200	2	102-118	8	0.567	0.549	- 0.034
417/418	2	178-204	11	0.754	0.809	0.068
464/465	2	130-152	10	0.587	0.610	0.038
EV1Pm	2	121-129	5	0.553	0.532	- 0.040
EV37Mn	2	192-224	17	0.900	0.923	0.026
EV94Mn	2	201-221	11	0.808	0.817	0.012
EV96Mn	2	183-215	17	0.854	0.866	0.014

### N_e_ estimates

The contemporary *N*_*e*_ estimated with NeEstimator ranged from 1,039 (*P*_*crit*_ = 0.01, 95% CI = 731 - 1,707) to 1,537 individuals (*P*_*crit*_ = 0.05, 95% CI = 754 - 18,851) for the different critical values. However, *P*_*crit*_ = 0.02 is indicated to provide better precision (Waples, 2006), therefore our more reliable estimate was 1,078 whales (95% CI = 738 - 1,884). From the above values the census population size (*N*_*c*_) was calculated as 3,880 individuals (95% CI = 2,656 - 6,782).

**Figure 3:**
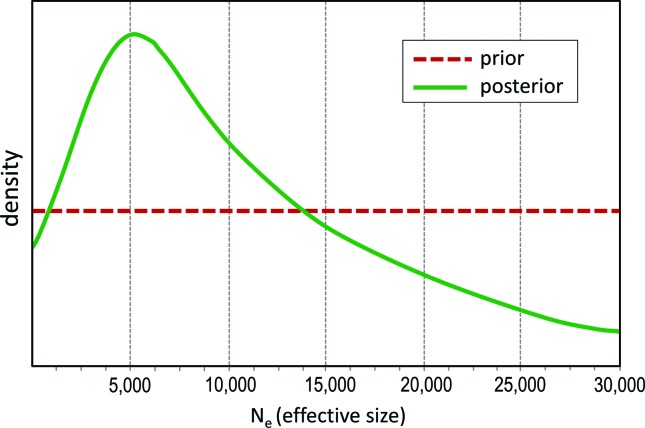
Posterior distribution (in green) of the parameter *N*_*e*_ from the best-supported scenario (Scenario 1, constant population, see Figure 2) as estimated in the program DIYABC. The red line is the prior distribution for *N*_*e*_.

In the comparison of the four scenarios (constant population, bottlenecked, expanded, and transitory bottleneck) using the ABC approach implemented in DIYABC, the constant population (no demographic changes) scenario was highly supported (posterior probability > 0.99) in relation to the other scenarios with demographic changes, in both the direct estimate and logistic regression approaches (Figure 2b,c). In the constant scenario, the mode of the posterior distribution for the long term *N*_*e*_ was 4,170 (95% CI = 2,330 - 26,600) (Figure 3). The mode for the *N*_*c*_ was then 15,012 (95% CI = 8,388 - 95,760), using the 3.6 census/effective ratio.

## Discussion

Our extended sampling confirms our previous results on the high nuclear DNA diversity of the humpback whale population that winters off the Brazilian coast (BSA), which is also compatible with its high mtDNA variability (Engel *et al.*, 2008; Cypriano-Souza *et al.*, 2010, 2017). This high genetic diversity is in agreement with other breeding grounds studied in the Southern Hemisphere for both nuclear and mitochondrial markers (e.g. Valsecchi *et al.*, 2002; Garrigue *et al.*, 2004; Pomilla and Rosenbaum, 2006; Olavarría *et al.*, 2007; Rosenbaum *et al.*, 2009; Cypriano-Souza *et al.*, 2017). Nevertheless, in the Southwestern Atlantic population, as well as most other humpback populations, severe reductions of their historical size by commercial whaling are well documented. The lowest number reached for breeding stock A was in the late 1950s, when only around 500 individuals (95% CI = 152 to 3,687) were estimated for this population (Zerbini *et al.*, 2011b). However, our previous study did not detect any significant signal of a genetic bottleneck in this population (Cypriano-Souza *et al.*, 2010), using three standard methods: heterozygosity excess (Cornuet and Luikart, 1996), mode-shift (Luikart *et al.*, 1998) and M-ratio tests (Garza and Williamson, 2001). That result was corroborated here with an extended data set and an ABC approach, in which by far the best supported scenario was a constant population compared with those in which a population experienced a single size change (expansion or bottleneck) or a bottleneck during the commercial whaling (between 2 and 8 generations ago) followed by an expansion (Figure 2).

As discussed previously, these results are consistent with the hypothesis (Amos, 1996) that the genetic bottleneck caused by commercial whaling was not strong enough to have left a significant signal in the BSA population (Engel *et al.*, 2008; Cypriano-Souza *et al.*, 2010). The magnitude of the genetic bottleneck is related to its duration and the minimum absolute size of the population during the bottleneck (Frankham *et al.*, 2002). In this population, large scale whaling lasted for only about four generations, assuming that the overexploitation of this stock was in the period between 1904 and 1967 (Paiva and Grangeiro, 1970) and a generation time of 18 years, taking into account the range between 12 and 24 years estimated for humpback whales (Chittleborough, 1965; Roman and Palumbi, 2003). In addition, the minimum absolute population size reached was relatively large (*N*_*min*_ = 500 individuals in the late 1950s) for the BSA population (Zerbini *et al.*, 2011b). Therefore, fewer generations with a not so small absolute effective population size should have left only weak genetic bottleneck signals and are, therefore, more difficult to detect. However, this estimation based on catch records did not incorporate missing whaling records between 1929 and 1946, a period in which the catch records in the breeding grounds are incomplete, producing likely biased estimates of depletion levels for this population (Morais *et al.*, 2017).

Recently, Phillips *et al.* (2012) showed a similar result with an ABC analysis for the bowhead whales (*Balaena mysticetus*), in which a bottleneck scenario was also not supported. In contrast, a recent ABC analysis for the Antarctic fur seal (*Arcthocephalus gazella*) supported a bottleneck scenario, although they have not detected a bottleneck using standard tests (Hoffman *et al.*, 2011). These different results were expected given the contrasting values for the two key parameters discussed above between the bowhead whale and the Antarctic fur seal: the generation length of the former was estimated ~50 years and for the latter ~10 years, while the minimum population size during the bottleneck for the former was around 1,000 individuals and for the latter it was as low as ~30-60 individuals (Phillips *et al.*, 2012). Interestingly, the inclusion of ancient samples may importantly increase the power to detect recent bottlenecks, as demonstrated by the study with the eastern Pacific gray whales (*Eschrichtius robustus*) (Alter *et al.*, 2012).

Although the methods used here assumed closed population with discrete generations (no generation overlap), random mating and equal contribution of individuals to the next generation, these assumptions are rarely completely satisfied in natural populations. The humpback whale is a long-lived species with overlapping generations, and some migration between breeding grounds in the Southern Hemisphere has been documented (Rosenbaum *et al.*, 2009). However, a recent study indicates that single-sample estimators of contemporary *N*_*e*_ based on linkage disequilibrium were not affected significantly with migration rates up to approximately 5–10% (Waples and England, 2011). Indeed, the migration rates between the Atlantic and Pacific breeding grounds of South America were estimated to be lower than the above limits (Cypriano-Souza *et al.*, 2017). Likewise, low migration rates based on mtDNA data have been estimated between humpbacks from Brazil and from breeding grounds in the Southeastern Atlantic Ocean (H.C. Rosenbaum, personal communication). The humpback whale is an age-structured species, and our estimates based on mixed-age samples reflect the effective size per generation (*N*_*e*_), but these estimates are approximately equal to population *N*_*e*_ when the sample includes as many cohorts as there are in a generation (Waples *et al.*, 2014). As we sampled only mature adults from nine different breeding seasons, the number of cohorts in this sample is lower than the generation length. However, our estimates should be fairly robust, albeit perhaps slightly lower, since the species is long-lived and has intermittent breeding (Waples and Antao, 2014).

Our *N*_*e*_ estimates with NeEstimator and DIYABC (point estimates 1,078 and 4,170 individuals, respectively) differed significantly. These differences can be explained mostly because these methods are expected to estimate *N*_*e*_ on different periods. The estimator from NeEstimator uses the amount of linkage disequilibrium within a population and is specifically designed to estimate *N*_*e*_ from the parental generation of the sample. In contrast, the *N*_*e*_ inferred from the ABC approach, considering the constant population scenario, is a long-term (that extends well before the whaling period) population size estimator, that represented the weighted harmonic mean of population sizes over 4*N*_*e*_ generations.

The most recent contemporary abundance estimates of the Brazilian humpback whale population, derived from aerial surveys that covered the entire stock range, estimated 6,404 individuals (95% CI = 5,085–8,068) in 2005, 7,689 individuals (95% CI = 6,585–8,931) in 2008, 8,652 individuals (95% C.I. = 7,696–9,682) in 2011, and 12,123 individuals (95% C.I. = 10,811–13,531) in 2015 (Andriolo *et al.*, 2010; Pavanato *et al.*, 2017). However, recent abundance estimates derived from ship surveys estimated 16,410 individuals (95% C.I. = 10,563–25,495) in 2008 (covered the total population range), and 19,429 individuals (95% C.I. = 15,958–23,654) in 2012, which were about 50% higher than that of the aerial surveys (Bortolotto *et al.*, 2016a). The difference between aerial and ship abundance estimates can be explained mainly by two sources of bias (underestimated group sizes and perception bias) of the first methodology, which may bias downwards the aerial abundance estimates. Therefore, assuming that our census size (*N*_*c*_) estimated by the NeEstimator, between around 2,600 and 6,800, corresponds to the parental generation of our sample (collected between 1999 and 2007), which given the uncertainty in the generation time would roughly correspond to the years between around 1980 and 1990. These values are broadly compatible with the abundance ship surveys extrapolated to the past using the growth rate of 7.4% per annum (Ward *et al.*, 2011).

The study on historical (pre-whaling) abundance of the stock A based on catch records using a Bayesian statistical method estimated the population size to nearly 24,700 individuals (95% CI = 22,804-31,220) before exploitation by modern whaling (Zerbini *et al.*, 2011b). Our point estimate for the long term *N*_*c*_ (~15,000) using the ABC approach was smaller than the pre-whaling abundance cited above, although the confidence intervals widely overlap that estimate. Furthermore, it is compatible with the most representative abundance estimate of approximately 16,000 humpback whales derived from shipboard survey in 2008 (Bortolotto *et al.*, 2016a). However, our results present broad confidence intervals usually derived from large uncertainties of several parameters, such as generation time, mutation rate, or the relation between *N*_*c*_ and *N*_*e*_. Consequently, more loci, more realistic scenarios (non-instantaneous population growth, gene flow, etc.) and methods are necessary to better estimate the demographic history of this population.

Overall, our results corroborate previous studies that have found high genetic diversity in the humpback whale population wintering off the Brazilian coast and no statistically significant reduction in its genetic diversity caused by modern whaling (Engel *et al.*, 2008; Cypriano-Souza *et al.*, 2010). Compatible with that, and estimated here for the first time, the *N*_*e*_ of the Southwestern Atlantic Ocean humpback whale population is relatively large. It has been suggested that the short-term minimum viable population size is *N*_*e*_ > 50 and the long-term minimum viable population size is *N*_*e*_ > 500 (Franklin and Frankham, 1998). Therefore, the contemporary *N*_*e*_ estimates of the Brazilian humpback population obtained here were above of these limits, suggesting good protection for its evolutionary future. However, we suggest that similar population size estimates should be carried out every 12 to 24 years (one generation) in order to obtain comparable *N*_*e*_ estimates and to monitor this population. To better accomplish this goal, future studies should attempt to reduce uncertainties of several key parameters, for example increasing the number of loci.
